# A comparison land-water environment of maximal voluntary isometric contraction during manual muscle testing through surface electromyography

**DOI:** 10.1186/2052-1847-5-28

**Published:** 2013-12-16

**Authors:** Romualdo Castillo-Lozano, Antonio Ignacio Cuesta-Vargas

**Affiliations:** 1 Faculty at Seville University, Seville, Spain; 2 Faculty of Health Sciences at Malaga University, Malaga, Spain; 3 School Clinical Science at Queensland University of Technology, Brisbane, Qld, Australia

**Keywords:** Surface electromyography, Aquatic exercise, Rehabilitation

## Abstract

**Background:**

The aim of this study was to compare through surface electromyographic (sEMG) recordings of the maximum voluntary contraction (MVC) on dry land and in water by manual muscle test (MMT).

**Method:**

Sixteen healthy right-handed subjects (8 males and 8 females) participated in measurement of muscle activation of the right shoulder. The selected muscles were the cervical erector spinae, trapezius, pectoralis, anterior deltoid, middle deltoid, infraspinatus and latissimus dorsi. The MVC test conditions were random with respect to the order on the land/in water.

**Results:**

For each muscle, the MVC test was performed and measured through sEMG to determine differences in muscle activation in both conditions. For all muscles except the latissimus dorsi, no significant differences were observed between land and water MVC scores (p = 0.063–0.679) and precision (%Diff = 7–10%) were observed between MVC conditions in the muscles trapezius, anterior deltoid and middle deltoid.

**Conclusions:**

If the procedure for data collection is optimal, under MMT conditions it appears that comparable MVC sEMG values were achieved on land and in water and the integrity of the EMG recordings were maintained during wáter immersion.

## Background

Electromyography (EMG) has emerged very recently in the area of aquatic physiotherapy (APT) [1]. This comes with the goal of applying new tools in people with some type of dysfunction due to any cause of muscle activation [2-4]. Early motion is critical to restoration of normal shoulder function. Aquatic therapy has been promoted as a method for increasing range of motion while minimizing stress on the shoulder [1]. Various water exercises exist for rehabilitation or fitness maintenance. In water, buoyancy acts against the body to reduce the load at the joints, while water viscosity requires the subject to exert greater force than when moving on land [4]. However, we note that there is a large knowledge gap in the welfare field associated with the dysfunction to the scapulohumeral rhythm and shoulder when we reviewed the scientific literature.

The surface electromyography (sEMG) has been an important tool in swimming in order to determine the muscle activity in propulsion [5-7]. However, in APT, Silvers & Dolny have used similar methodologies for the validation of sEMG on dry land/in water through the MVC test [8]. In this study we observed that under MMT conditions it appears that comparable MVC sEMG values were achieved on land and in water and the integrity of the EMG recordings were maintained during water immersion [8]. The degree of muscle activation during aquatic exercise has become a challenge for the APT today [9-11]. In this study, the main differences from Silvers & Dolny are the randomization of the sample and the measurements of shoulder musculature.

Therefore, in this study a MVC test of the shoulder muscles is performed both in water and dry land, to determine the degree of comparison, integrity, validity and reliability of sEMG signal in both environments, considering what Silvers & Dolny said on the importance of using sEMG waterproofing procedures [8].

## Method

### Participants

We studied the musculature of the right shoulder of 16 skilled and healthy subjects (8 men and 8 women) upon acceptance of voluntary informed consent. They had a mean age of 26.06 ± 4.48 years. All subjects were interviewed using the questionnaire International Physical Activity Questionnaire [12] and measured anthropometric criteria according to International Society for the Advancement of Kinanthropometry [13] according to the standards of the Committee on Human Experimentation of the institution in which the experiments were done or in accordance with the Helsinki Declaration of 1975.

### Preparation and placement of sEMG

**Figure 1:**
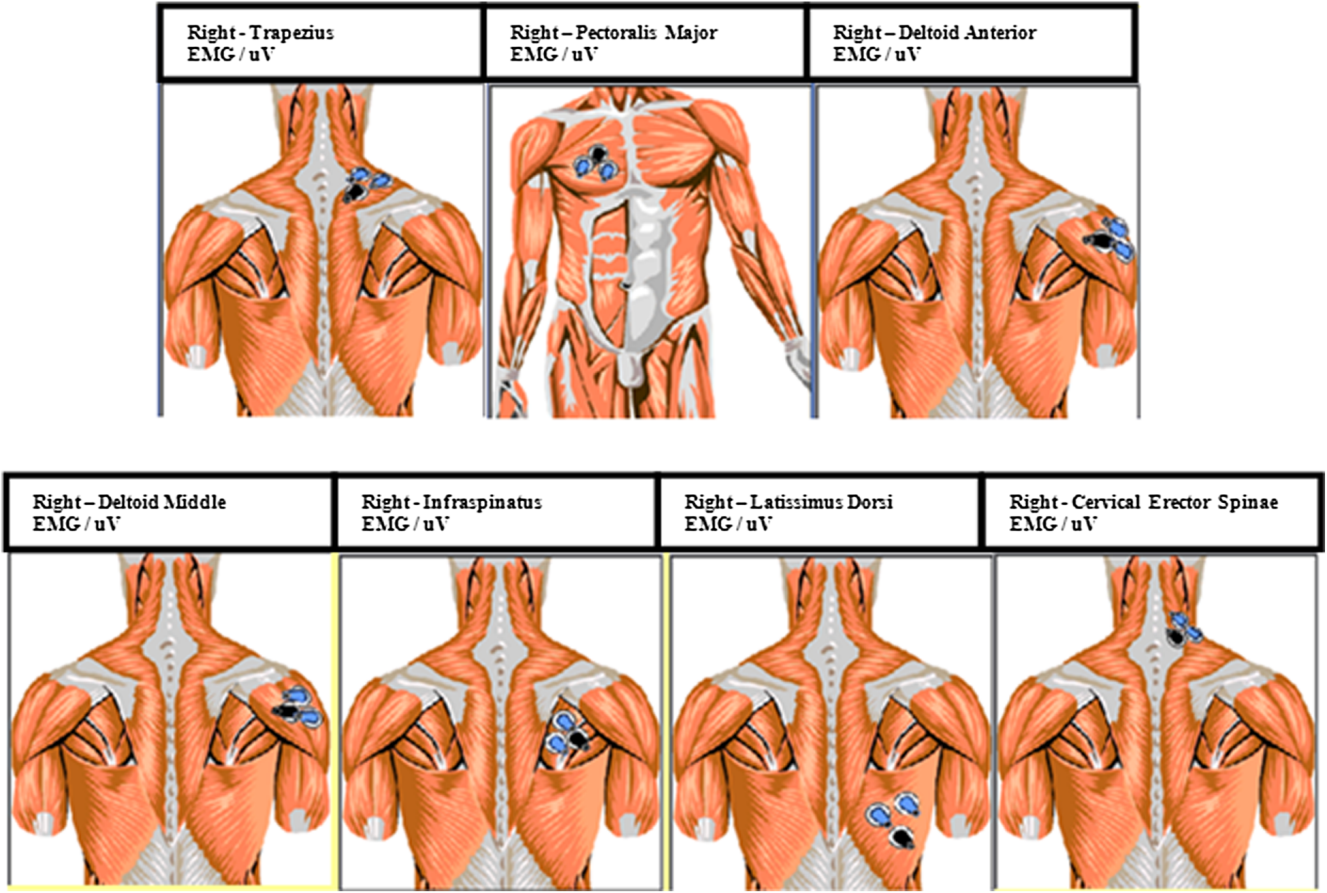
Electrode placement according Megawin 3.0.1. (Mega Electronics Ltd, Kuopio, Finland).

Adhesive surface electrodes (5mm diameter) were used on all subjects after skin cleaning with a 90% alcohol swab, application of an adhesive spray (TensoSpray™) and protection by a waterproof adhesive tape (Tegaderm, 3M, St Paul, US) [14]. This was previously validated in a pilot study and recommended for experimental use, where we found that the electrode waterproofing procedures and the signal to be identical in both environments. The sEMG signals were visually assessed prior to MVC testing using maximal effort MMT to ensure accurate electrode placement. Lastly, all testing procedures were performed by the same researcher to improve consistency of electrode placement. Electrodes were separated by 1-3 cm of muscle according to the method described by Surface Electromyography for the Non-Invasive Assessment of Muscles (SENIAM) [15] and placed on the seven muscles investigated: cervical erector spinae, upper trapezius, pectoralis major (sternal fibres), anterior deltoid, middle deltoid, infraspinatus and latissimus dorsi. The electrodes were placed on the muscle belly of each muscle, whose activity was measured by means of the Mega Win 3.0.1 software (Mega Electronics Ltd, Kuopio, Finland). We selected these muscles because Drake et al., [16] said they were the most important in the stabilizing and the mobility of shoulder (Figure 1).

### MVC test

The MVC test was used to verify the integrity and comparability of the sEMG signal on dry land/in water. This test was completed in accordance with the recommendations of the Perotto [17] MMT test consisting of three repetitions of the MCV test of 5 seconds each, separated by at least 30 seconds of recovery, with neutral arm rotation and no movement (isometric force) on dry land/in water. The subject’s position was identical in both environments (anatomical position reference). This was done on dry land/in water in order of randomization, and it was reinforced by visual and verbal encouragement. The water depth was adjusted to the edge of the acromion for each MVC test. The order for the data collection was identical for each MVC test on dry land/in water (Table 1).

**Table 1:** Execution of MMT test for subsequent data collection with sEMG

**Cervical erector spinae**	The therapist stands behind the participant and resists neck extension.
**Trapezius**	The therapist stands behind the participant and unilateral action resists shoulder elevation and tilt of the head.
**Infraspinatus**	The physiotherapist is placed laterally to the participant and resists external rotation and separation of the arm, elbow flexion of 90°.
**Anterior deltoid**	The therapist is positioned before the participant and resists forward flexion and internal rotation of the arm.
**Middle deltoid**	The physiotherapist is placed laterally to the participant and resists separation of the arm to 90 degrees.
**Pectoralis**	The therapist is positioned before the participant and resists the approach and internal rotation.
**Latissimus dorsi**	The therapist stands behind the participant and resists the approach and arm extension, and internal rotation.

### Acquisition and processing of data

During the MVC test, sEMG data was recorded at a sampling frequency of 1000 Hz. A low pass filter was applied with a bandwidth of 20 Hz, an attenuation of 60dB and a maximum frequency of 400 Hz.

### Statistical analysis

Data was analysed with SPSS version 15 for Windows. Following the intervention phase, we continued the inferential analysis between variables, according to type and normality. For the analysis of the variables, we performed the nonparametric Kolgomorov-Smirnov test and used Wilcoxon or Student’s t-test for the normality of the variables. Finally, the ICC and the Cronbach's alpha tests were used to calculate correlation between both measurements. The statistical significance level was set at 0.05.

## Results

**Figure 2:**
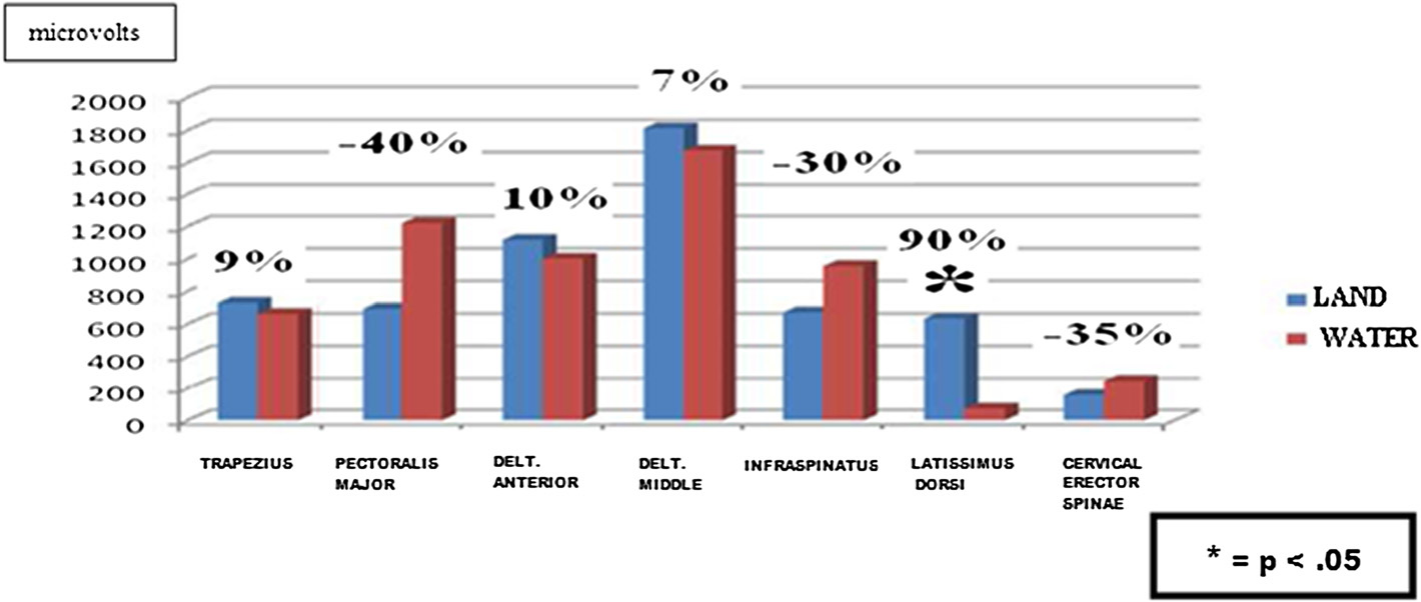
Analysis through sEMG for MVC test in the different muscles in land/water.

As can be seen in Table 2 and Figure 2, there were no statistically significant differences between means, with the exception of the latissimus dorsi MVC test on land/in water. However, the trapezius, anterior deltoid and middle deltoid presented as means very close in both environments.

**Table 2:** Analysis through sEMG for MVC test in the different muscles in land/water

**LAND**	**MAX_ISO_TRAP**	**MAX_ISO_PECT**	**MAX_ISO_DELTA**	**MAX_ISO_DELTM**	**MAX_ISO_INFR**	**MAX_ISO_DORS**	**MAX_ISO_ESPIN**
**Average(μV)**	728.37	693.37	1116.00	1812.12	671.43	633.06	156.87
**SD**	±570.17	±433.43	±705.02	±1079.56	±450.13	±479.32	±128.50
**95% Confidence Intervals**	424.54	462.41	740.31	1236.86	431.57	377.64	88.39
	1032.20	924.33	1491.68	2387.38	911.29	888.47	225.35
**WATER**	**MAX_ISO_TRAP**	**MAX_ISO_PECT**	**MAX_ISO_DELTA**	**MAX_ISO_DELTM**	**MAX_ISO_INFR**	**MAX_ISO_DORS**	**MAX_ISO_ESPIN**
**Average(μV)**	661.75	1219.12	1000.12	1674.37	954.81	72.00	247.06
**SD**	±636.55	±1011.54	±821.70	±2656.80	±675.07	±117.34	±348.19
**95% Confidence Intervals**	322.55	680.10	562.26	258.66	595.08	9.47	61.52
	1000.94	1758.14	1437.98	3090.08	1314.53	134.52	432.60
**Difference% land/water**	9%	−40%	10%	7%	−30%	90%	−35%
**ICC**	.278	−.004	−.009	.024	.151	.100	.082
**Cronbach**	.435	−.009	−-017	.047	.263	.182	.152
**Sig.**	.134	.063	.679	.148	.301	.001*	.569

*= p < .05μV: microvolts.

ICC: intra-class correlation coefficient.

SD: Standard Deviation.

MAX_ISO_TRAP: Maximum isometric trapezius.

MAX_ISO_DELTA: Maximum isometric anterior delt.

MAX_ISO_PECT: Maximum isometric pectoralis.

MAX_ISO_DELTM: Maximum isometric middle delt.

MAX_ISO_INFR: Maximum isometric infraspinatus.

MAX_ISO_DORS: Maximum isometric latissimus dorsi.

MAX_ISO_ESPIN: Maximum isometric cervical erector spinae.

## Discussion

### Comparison of scores through sEMG of the MVC test on land/in water

There is a body of evidence from underwater sEMG recording that demonstrates reduced signal amplitudes and decreased sEMG/force ratios for maximal isometric muscle contractions compared with measurements in air that would question the results of the present study [8]. Some studies conclude that the MVC test in water has less in signal amplitude with respect to the test on land [18-21]. Pöyhönen et al. [16] observed that MVC force output and muscle activity of the quadriceps significantly decreased during isometric knee extension exercise in water vs. land [18]. Pöyhönen et al. [17] observed significantly decreased sEMG amplitudes during water MVC testing [19]. Additionally, Pöyhönen & Avela [18]; and Pinto et al., [22] reported that, compared to land, isometric plantar flexor MVC output was approximately 13% lower during water immersion inducing a deterioration of neuromuscular function, perhaps by triggering inhibitory mechanisms. The origin of these mechanisms seems to be related mainly to effects of partial weightlessness and the hydrostatic pressure [20,22]. In our study, three of the seven muscle-measured results show less than 10% regarding in muscle activation in the MVC test in water compared to dry-land (trapezius, anterior deltoid and middle deltoid), although such differences did not reach statistical significance.

The present study did not attempt to elucidate the neurophysiological factors that have been suggested to explain the differences in sEMG recordings between land and water. Instead, we focused on reducing the errors in the procedure for the sEMG recordings so that land and water MVC tests could be compared more accurately. These minimal differences were attributed to the sealing of the electrodes [14,19] and the participant’s motivation during measurement through sEMG of the MVC test, execution and level of resistance in the MMT test for investigator. We agree with Silvers & Dolny [8] about the importance of waterproofing, but we believe that the motivation of the participant and the examiner’s resistance may be an important factor to consider because it is impossible to say with certainty that the difference between the two environments in the MCV test is exclusively due to poor waterproofing. In our study, we focused on reducing the influence of water infiltration on the sEMG recordings, so that land and water MVC tests could be compared more accurately. Visual artifacts (high frequency in the sEMG signal) during water submersion were not observed during MVC testing. Significant differences between the MVC scores from Land and Water also were not observed, except in the latissimus dorsi (p = 0.001). We suspect that the cause of the difference is due to the implementation of the MVC test, which was not 100%, or to the fact that the resistance in MMT test was not appropriate in water [14,18-20].

### Reproducibility of the measurements through sEMG of the MVC test on land/in water

To evaluate the reproducibility of the peak was used in the MVC test for each muscle in both conditions [8,14,22-24]. The reproducibility of the recordings was considered high in the trapezius, anterior deltoid and middle deltoid (%Diff = 7–10%), coinciding with other studies, such as that of Silvers & Dolny (%Diff: 7.4–12.6%) [8]; Pöyhönen et al. (%Diff = 3.5–11%) [18] or Pinto et al. (%Diff: 29–35%) [22].

Also observed in the muscles pectoralis, the cervical erector spinae and infraspinatus muscles produce more electrical potential in the aquatic environment on land. This occurs because of the complexity of the MVC test in two environments as different as water and land, because the thrust of the participant and the examiner’s resistance (MMT) is clearly influenced depending on the environment. Although initial tests (pilot study) performed where we found that the waterproofing were absolute and applied low-pass filters to prevent water-noise inferences; we can say that repeating the same procedure for the maximum thrust of the participant and offer the same resistance to the participant in both environments, it is very difficult and complex task.

Finally, the correlations according to intra-class correlation coefficient (ICC) and alpha Cronbach test between the two environments are not very linear relationships in the pectoralis, anterior deltoid and middle deltoid, as the values are very close to zero. Others muscle (trapeze, latissimus dorsi, infraspinatus and erector spine neck) have a higher degree of positive relationship between them and values away from zero. In general, we say that the relationships between measurements in both environments and the application of the procedure by MVC test measured with sEMG are very weak because the sample is heterogeneous, participants possessed very different anthropometric values and mediation in water (buoyancy) is more complex than on land. The application of randomization tests on dry land/in water interventions had no effect [14,18-20,22-24].

### Limitations

The current study has several limitations. Participants were provided verbal encouragement throughout testing. To facilitate a MVC test, participants were positioned in identical anatomical conditions on land and in water. MMT was used for each MVC test, which may have introduced variation in resistance provided to the limbs. We recognize that the use of external resistance, such as firmly secured mobilization straps, may have facilitated reliability for generating a MVC. Water temperature could have affected the sEMG recordings in this study. However, water temperature was approximately 29–30ºC, reducing the likelihood that temperature distorted the sEMG signal [25]. Veneziano et al. [16] suggested that several factors may contribute to the discrepancy in the literature regarding muscle sEMG recordings in water [25]: (1) the adoption of different protocols; (2) water leakage to the electrodes that causes changes in the sEMG variable estimate (the crosstalk [3]); (3) the study of different muscles [14]; (4) the role of buoyancy forces; (5) different degrees of body immersion, from the isolated limb to the whole body; and (6) different temperatures of the water with respect to the skin. Our results provide evidence that proper protection of electrodes and leads will solve the water leakage issue.

Therefore, the degree of immersion and variety of muscles tested still appear to be factors that may partially account for the disparate results of previous research.

## Conclusion

We know that the reproducibility of a well-described procedure in the same environment is complicated by numerous factors endogenous and exogenous to the participant/researcher in each of the environments. When comparing the same procedure on land and in water, endogenous and exogenous changes to the participant/researcher are much higher, so it is very difficult to reproduce the procedure exactly as in water/on land. However, following the methodological rigor taken in this study and the accompanying data analysis, we can say that the use of the test protocols described for MVC test, the optimal application of MMT test in water/on land, the maximum force of participant at the MVC test and the waterproofing, which does not allow the entry of water between the electrode/skin, all appear to make the values of the MVC test for sEMG more comparable with that on dry land/in water.

## Abbreviations

sEMG: surface electromyographic; MVC: Maximum voluntary contraction; MMT: Manual muscle test; APT: Aquatic physiotherapy; μV: Microvolts.

## Competing interests

The authors declare that they have no competing interests.

## Authors’ contributions

AIC-V participated in the design of the study and performed the statistical analysis and to drafted the manuscript. RC-L collected the data and helped to draft the manuscript. Both authors read and approved the final manuscript.

## Authors’ information

PhD candidate at University of Malaga and Lecturer of Physiotherapy of Faculty of Health Sciences at Osuna Extension University.

## Pre-publication history

The pre-publication history for this paper can be accessed here:

http://www.biomedcentral.com/2052-1847/5/28/prepub
